# Determinants of Human Asymmetry: Does Asymmetrical Retinal Vasculature Predict Asymmetry Elsewhere in the Body?

**DOI:** 10.3390/life14080929

**Published:** 2024-07-24

**Authors:** Doris Plećaš, Vesna Gotovac Đogaš, Ozren Polašek, Jelena Škunca Herman

**Affiliations:** 1 University of Split School of Medicine, 21000 Split, Croatia; doris.plecas@gmail.com; 2 University of Split School of Mathematics, 21000 Split, Croatia; vgotovac@pmfst.hr; 3 Croatian Science Foundation, 10000 Zagreb, Croatia; 4 Sisters of Mercy Clinical Hospital, 10000 Zagreb, Croatia

**Keywords:** asymmetry, retina, fitness, survival, human

## Abstract

The aim of this study was to explore retinal vasculature asymmetry (ReVA) patterns in subjects from the islands of Vis and Korcula and the city of Split, Croatia. Asymmetry estimates were based on topographic image analysis of non-mydriatic retinal fundus photographs and compared with nine ophthalmic measurements, three Doppler-based pressure indices and eight frequencies of audiometry. ReVA was also correlated to the genomic runs of homozygosity (ROHs) and used in a Cox regression survival model, where we adjusted for the effects of sex, age and comorbidity. In 1873 subjects, ReVA estimates were significantly correlated with most ophthalmic asymmetry measures, less strongly with the ankle–brachial pressure index and only modestly with higher-amplitude audiometry asymmetries (lowest *p* = 0.020). ReVA was significantly correlated with the number of ROHs (r = 0.229, *p* < 0.001) but less strongly with the ROH length (r = 0.101, *p* < 0.001). The overlap of asymmetries was low, with only 107 subjects (5.7% of the total sample) who had two or more instances in which they were among the top 10%. Multiple asymmetries did not affect survival (HR = 0.74, 95% confidence intervals 0.45–1.22). Retinal vasculature asymmetry is a poor predictor of asymmetry elsewhere in the body. Despite its existence and apparent association with comorbidities, the observed extent of retinal vasculature asymmetry did not affect the lifespan in this population.

## 1. Introduction

One of the fundamental questions in developmental biology is of the determinants of asymmetries in body growth and development [1]. The differences in bilateral body elements can be described as fluctuating asymmetry, directional asymmetry and antisymmetry [2]. Fluctuating asymmetry shows a balanced situation with the left–right difference averaging to zero; directional asymmetry shows a skewness to one side; while antisymmetry describes two clusters of the population that produce an average of zero [2].

The underlying causes for asymmetries suggest that they can originate due to genomic and environmental effects, predisposing organisms to develop unequal left and right body sides [3,4,5,6]. Furthermore, human bodily symmetry has been shown to alter from a comparatively more asymmetric childhood, reaching an optimum in adulthood and subsequently increasing in senescence [7,8,9]. However, the margin of error in estimating asymmetries is very challenging, questioning theory in practice [10,11,12], suggesting, at best, only modest evidence of an association of asymmetry with fitness- and survival-linked phenotypes [13,14,15].

Genomic measures can also be used in relation to phenotypic asymmetry. One of the basic approaches is the use of genomic inbreeding estimates [16,17], which are gradually being replaced by runs of homozygosity (ROHs) [18], a measure that informs us about the autozygous stretches of DNA that denote a homozygous sequence that is identical by descent [19]. Such estimates are particularly interesting in isolated populations, which retain specific genetic structuring with a high share of endogamy and may be comparatively more useful in gene mapping [20]. Notably, interpreting the results linked to ROHs must be performed cautiously since numerous demographic and historical processes may affect them [21,22,23]. Despite many published studies that were likely underpowered to detect a real effect, a large-scale consortium has confirmed the adverse effects of increased homozygosity for some human traits, like height and cognition [24].

Asymmetry is critical in organs that integrate the left and right body sides, especially the eye. Even a small extent of asymmetry can cause functional problems in certain parts of the eye. Interestingly, a previous study has already linked some forms of ophthalmic function reduction in situations of higher ROHs [19]. Despite previous difficulties asserting retinal phenotypes [25,26], recent studies have often employed more advanced image analytic methods that enable quicker and automated analysis [27,28,29,30,31].

This study’s aim was to pursue multiple objectives. First, it aimed to investigate the patterns and distribution of retinal blood vessels. Next, we explored the correlation between retinal asymmetry and asymmetry observed in various other ophthalmic measurements, and its correlation with body asymmetry. Last, the investigation extended to examine whether ROHs, a fundamental genomic metric, could be linked to retinal blood vessel asymmetry.

## 2. Materials and Methods

This study was based on the 10.001 Dalmatians project, which aimed to explore the genetic and environmental risk factors for health and disease in isolated human populations [20]. These included the inhabitants of two remote islands, the islands of Vis (*n* = 617) and Korcula (*n* = 470), complemented by subjects recruited from the mainland in the coastal city of Split (*n* = 786). All subjects were first informed about the study goals, risks and procedures, after which they signed informed consent before inclusion. The ethics boards of the Medical School, University of Zagreb, the Multi-Centre Research Ethics Committee for Scotland, The University of Split School of Medicine and the Lothian NHS Board approved this study.

For the purposes of this study, we only included a subset of the subjects who met two inclusion criteria. First, the subjects were selected based on whether we could access their bilateral retinal fundus photographs for analysis and whether these photographs were of sufficient quality to perform the assessment. Secondly, only subjects with available DNA genotypic data were included. All subjects filled out a detailed survey, which was used, in part, for this study. Of note, we included data related to the socioeconomic status of participants (except for the objective socioeconomic status estimation from the Vis sub-cohort).

### 2.1. Clinical Data

All subjects underwent a series of examinations and measurements, some of which were used in this study. The ankle–brachial pressure index (ABPI) is a simple measurement based on Doppler attenuation and a sphygmomanometer (Mini Dopllex D900, Huntleigh, Cardiff, UK). The subjects were asked to lie calmly for at least 10 min before the recording, followed by a brachial systolic blood pressure measurement on both sides. The same protocol was performed for the posterior tibial and dorsal pedis arteries, yielding a total of three bilateral measurements (six measurements in total). For this study, we did not calculate the ABPI as the ratio of the legs and arms; instead, we used the bilateral systolic measurements to infer asymmetries. All ABPI measurements were performed by two licensed MDs, with weekly quality control assessments.

A total of nine ophthalmic measurements were used to infer ocular asymmetry. These were based on keratometry and noncycloplegic autorefraction, measured in both eyes with a hand-held autorefractometer/keratometer (Ark30; Nidek, Gamagori, Japan). In addition, we used biometry measurements with an A-scan device (Echoscan US-1800; Nidek). For the A-scan, which required contact with the cornea, oxybuprocaine anaesthetic sterile eye drops (Minims; Chauvin Pharmaceuticals, Ltd., Romford, UK) were used. All measurements were made by licenced ophthalmologists. In total, nine measurements were used in the analysis, including the spheric power, cylinder power, angle, corneal radius, corneal thickness, anterior chamber length, lens thickness, posterior chamber length and axial length.

We used analogue pure-tone audiometry, with thresholds of 0.25, 0.5, 1, 2, 3, 4, 6 and 8 kHz, to measure the hearing thresholds, measured on an analogue AD226 audiometer (Interacoustics, Middlefart, Denmark). The measurements were made in a quiet room, with a random selection of measurements, favouring the left or right ear for the first measurement. The procedure included a mid-frequency tone first played to teach the subjects what they expected to hear. Next, we browsed from low to high frequencies and recorded the hearing thresholds for every frequency. All subjects who reported traumatic events or unilateral causes of hearing loss (*n* = 59) were removed from the analysis.

We also defined a comorbidity load as the equally weighted sum of all known chronic diseases a subject reported or had in their medical history. Based on the available data, the values of this variable ranged from zero (denoting an apparently healthy subject) to a maximum of five observed concomitant diagnoses. We also split these data into three groups, denoting individuals with no known comorbidities, those with only one and those with multiple comorbidities, and used this in the analysis.

### 2.2. Retinal Fundus Photographs

All retinal fundus photographs were taken with a fixed digital fundus camera, Canon CR-DGi, with an attached EOS 30D camera. The recording was based on a non-mydriatic protocol completed in a dark room after about 10–20 min of sitting, during which the eyes could adapt to complete darkness to achieve maximum image quality. All photographs were taken by licensed ophthalmologists, who assessed the photograph quality immediately after recording and, in some cases, repeated the process after sufficient re-adaptation of the eye. In several cases, retinal fundus photographs were removed from the analysis due to poor image quality (*n* = 23) or a lack of bilateral images (*n* = 9).

### 2.3. Retinal Vasculature Asymmetry Estimates

To measure the asymmetry, we developed an analytical approach based on topological image analysis. Topology is a mathematical field that studies the properties of invariant objects under continuous deformations (like twisting and stretching but not tearing or merging). Topological data analysis (TDA) allows for the investigation of the properties of datasets using their topological features, deriving concepts from the data filtration, i.e., a parametrised family of topological objects, which increases the logic to include the parameter [32,33]. Persistent homology tracks how the topological features of the objects in the filtration persist as the parameter changes. Homology is a method for associating a sequence of algebraic objects with other mathematical objects, such as, in our case, topological spaces. Homology groups were introduced to compare two shapes based on their *k-*dimensional holes. The 0-th homology group *H*_0_ is constructed based on the objects’ connected components (zero-dimensional holes), the 1-th homology *H*_1_ corresponds to loops or one-dimensional voids and the 2-th homology *H*_2_ to voids or two-dimensional holes. Persistent homology captures the changes in these homology groups as the parameter changes by tracing the parameters of filtration when the *k*-dimensional holes appear and disappear. The structure of the persistence homology can be depicted via persistence diagrams or barcodes. In our application, we used persistence diagrams.

The *k*-dimensional persistence diagram (PDk) consists of the points whose coordinates are the parameter values of when the *k*-dimensional hole first appeared (referred to as the birth time of the feature) versus the parameter values when the *k*-dimensional hole disappears (referred to as the death of the feature), respectively. Therefore,

*PDk* = (*b_i_*, *d_i_*), *i* = 1, …, *n* (1)

where *b_i_* is the time of birth and *d_i_* is the time of death of the *i*-th feature. PDs are often transformed to the rotated and rescaled persistence diagram (RRPD), defined by

*RRP D_k_* = (*m*_1_, *l*_1_), … (*m_n_*, *l_n_*) (2)

where *m_i_* = (*b_i_* + *d_i_*)/2 stands for the mean age, and *l_i_* = *d_i_* − *b_i_* represents the lifetime of the *i*-th feature. Then, the accumulative persistence function (APF) can be defined as the summary function

*APF_k_*(*m*) = Σ*l_i_* 1(*m*_i_ ≤ *m*) (3)

where 1() denotes the indicator function [34]. The APF cumulatively sums the lifetimes of the features concerning their mean age. For the purposes of this study, and in conjunction with a previous similar approach [35], fundus photographs were first transformed to greyscale versions so that they could be interpreted as a two-dimensional field of values *z*(*i*, *j*) on an integer lattice of pixels *I*_(*i*,*j*)_, *(i*, *j*) ∈ [1, …, *N*] × [1, …, *M*]. 

The filtration was constructed by filtering out all of the pixels with an assigned value under the particular threshold level l. This yielded a non-decreasing family of cubical complexes, i.e., finite unions of cubes (pixels) with vertices in an integer lattice, from which we focused on 0-th and 1-th homology.

The cubical persistent homology was calculated in the programming language Julia using the *ripserer* package, v0.14.7 [36]. The obtained PDs were transformed into corresponding RRPDs to calculate the APF0 and APF1 for the left and right fundus photographs. Next, we used the difference between the APFs with the maximum absolute value for the asymmetry measurement. It can be viewed as a signed supremum metric between the APFs. Under the assumption of entirely symmetric photographs, their topological features should be the same since they are invariant under symmetry; therefore, the measure of asymmetry will be 0. As the asymmetry grows, the topological features should increasingly differ, leading to greater asymmetry measure values. In addition to just the absolute value of the extent of asymmetry, we also aimed to infer the existence of the directional asymmetry; therefore, we also retained the signed estimate. In more detail, we denoted, by *APFk_u,R_* and *APFk_u,L_*, the *APFk* obtained from the right and left images that were unit transformed, respectively, and the *APFk_z,R_* and *APFk_z,R_* that *APFk* obtained from the right and left images that were *z*-transformed, respectively. Therefore, we can define the following formulae:

ReVA.0 = *APF*0*_u,R_*(m_0,*max*_) − *APF*0*_u,L_*(m_0,*max*_), (4)

where m_0,*max*_ = argmax_m_(*APF*0*_R(m)_* − *APF*0*_L_*_(*m*)_). (5)

ReVA.1 = *APF*1*_R_*(m_1,*max*_) − *APF*1*_L_*(m_1*,max*_), (6)

where m_1,*max*_ = argmax_m_(*APF*1*_R_*_(*m*)_ − *APF*1*_L_*_(*m*)_). (7)

**Figure 1:**
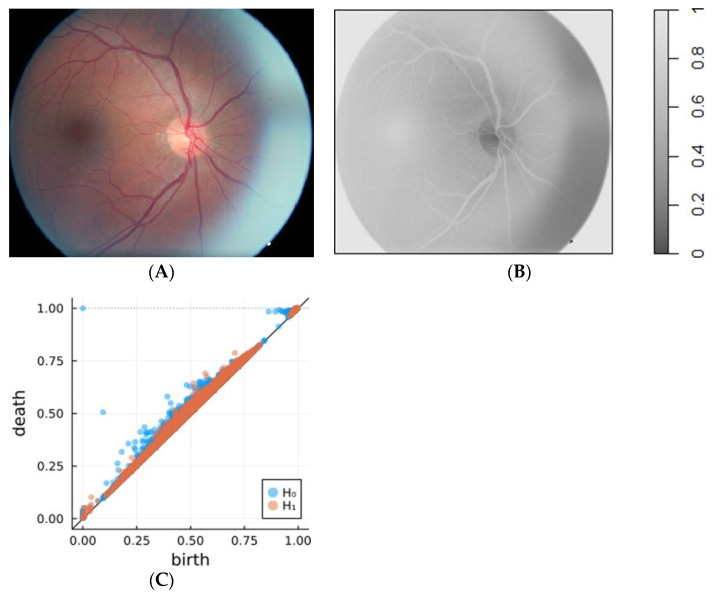
Steps in calculating the asymmetry coefficient between left and right fundus images. (**A**) An original colour photograph, (**B**) greyscale image, (**C**) persistence diagram for one eye.

Therefore, the protocol for the ReVA assessment was based on using the trimmed and aligned photographs (Figure 1A), which were first converted into a greyscale image (Figure 1B). Next, persistence diagrams were made (Figure 1C).

### 2.4. Genotyping and Runs of Homozygosity

All subjects provided a blood sample, which was used to extract DNA. The extraction was performed using Nucleon kits (Tepnel, Manchester, UK), followed by genotyping using the Illumina HumanHap300 array with 317,503 single-nucleotide polymorphisms (SNPs) (Vis sub-cohort) or Illumina HumanHap 370CNV (Illumina, San Diego, CA, USA; Split and Korcula sub-cohorts), with a total of 346,027 SNPs. All markers with a call rate of <98%, a minor allele frequency of <2% or out of Hardy–Weinberg equilibrium (*p* < 10^−10^) were removed in quality control.

Runs of homozygosity were calculated in PLINK (ver. 1.0) [37], with a moving window of 5000 kb. Three estimates were calculated: the number of ROHs, their length in kilobase (kb) and their average length in kb. In addition, we also performed a linkage disequilibrium (LD)-pruned dataset since LD may affect ROH estimates in isolated and small populations. For that purpose, we used MASEL [38], which selects a set of markers based on LD while maximising marker information content and genome coverage. An LD threshold of r2 ≤ 0.1 was used.

### 2.5. Statistical Analysis

In order to measure the asymmetries in a comparable way to ReVA estimates, we calculated several estimates. First, to assess the difference between the left and right sides, we used residuals, defined as the difference between the average values of the two bilateral measurements. Next, to assess the extent of asymmetry, we used the absolute value of this residual. Last, all of the asymmetry estimates were normalised to allow for averaging and enable direct comparisons, i.e., each value was divided by the maximum value for that variable. In this way, we obtained positive asymmetry estimates, which ranged from 0 to 1, were unitless and were directly comparable.

In the subsequent analyses, we focused on four body parts: the retina (the asymmetries based on ReVA.0 and ReVA.1), ophthalmic measures, three measures of ABPI and audiometry. Since we calculated normalised values, we could average across each group to obtain averaged symmetry. For all four situations, we classified all subjects into two groups: those belonging to the top 10% of the asymmetries and the rest. This way, we obtained four binary variables to address the primary hypothesis. The pairwise group overlap is shown in a Venn diagram.

Numerical data are shown as means and standard deviations, while categorical data are shown as numbers and percentages. Similarly, bivariate statistical analysis was based on the t-test for numerical and ANOVA for sub-cohort-based analyses, while categorical data were analysed with the chi-square test. Correlation analysis was based on Pearson’s test. In order to control the detected confounding effects, we used Cox regression in survival analysis, adjusting for the known and most likely confounders (including age, sex, cohort, the existence of multiple asymmetries in the same subject, comorbidities and runs of homozygosity). All analyses were performed with R (www.r-project.org), with significance set at *p* < 0.05.

## 3. Results

This study included 1873 subjects from three sub-cohorts (Table 1). There were 739 (39.5%) men in the total sample, with the lowest share in the island of Vis and the highest in the island of Korcula sub-cohorts (Table 1). Even the most basic comparison indicated numerous differences in the three sub-cohort demographics (Table 1).

The initial analysis showed that ReVA.0 and ReVA.1 were not significantly different in men vs. women (0.75 ± 0.19 vs. 0.76 ± 0.18, *p* = 0.128; 0.75 ± 0.19 vs. 0.76 ± 0.18; *p* = 0.138). In contrast, the difference across sub-cohorts was significant, with the highest values from the island of Vis (0.87 ± 0.10), intermediate on the island of Korcula (0.74 ± 0.17) and the lowest in the city of Split (0.68 ± 0.20; all *p* < 0.001). Interestingly, ReVA was uncorrelated with age (r = 0.01, *p* = 0.653 for ReVA.0 and r = −0.003, *p* = 0.913 for ReVA.1). All estimates of socioeconomic status were inversely and significantly correlated with ReVA, except objective material status, which had a marginally insignificant result (r = −0.05, *p* = 0.080 and r = −0.05, *p* = 0.077).

The correlation between asymmetry estimates indicated that the ReVA estimates were correlated with most ophthalmic measures, less strongly with ABPI, and the least strongly with the audiometry asymmetry estimates (Table 2). Notably, both ReVA measurements were significantly correlated with all of the ROH estimates, regardless of the LD pruning (Table 2).

**Figure 2:**
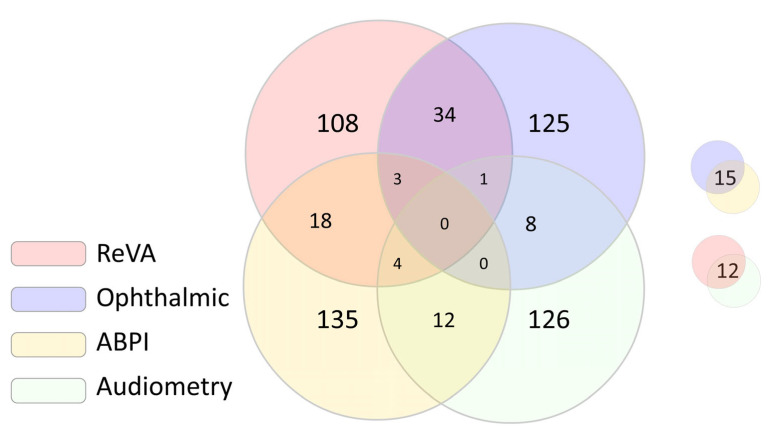
Venn diagram of the overlap between the top 10% of subjects according to the body part asymmetry.

The overlap analysis among the top 10% of most asymmetric subjects indicated that no subjects were asymmetric in all four analysed body parts (Figure 2). Furthermore, 8 subjects had three asymmetries, while an additional 99 had at least two asymmetries among the top 10% most asymmetric participants (Figure 1). Therefore, 107 subjects were found to have two or more asymmetries among the top 10%. These subjects were evenly distributed in terms of sex (6.8% of men vs. 5.5% of women; *p* = 0.280), but they were the most common on the most endogamous island of Vis (77; 12.8%), less common on the island of Korcula (23; 5.6%) and the least common in the city of Split (7; 0.9%; *p* < 0.001). They were, on average, older (56.7 ± 13.3 vs. 53.0 ± 14.6 years; *p* = 0.011) and had more comorbidities (58.9% vs. 47.5%, *p* = 0.023).

The survival analysis indicated that subjects with multiple asymmetries did not have significantly higher hazard ratios in an unadjusted model (Table 3). Adding the additional confounders to the adjusted model changed some estimates, but the result remained insignificant for the multiple asymmetries, suggesting they had no contribution to survival (Table 3). We then performed the same analysis stratified by sex. The results for both of these models suggested no difference in both estimates (HR = 0.97 [0.57–1.63]; *p* = 0.898 for ReVA and 0.99 [0.96–1.03]; *p* = 0.647 for ROHs, number of segments for men and HR = 0.96 [0.55–1.67]; *p* = 0.882 and 0.99 [0.96–1.03]; *p* = 0.599, for women, respectively).

Re-running the model with ReVA estimates as continuous variables instead of multiple asymmetries suggested that neither had a significant contribution to survival (HR = 1.00 [0.42–2.41]; *p* = 0.994 for ReVA.0 and HR = 1.06 [0.43–2.55]; *p* = 0.898 for ReVA.1).

## 4. Discussion

The results of this study indicate that retinal vasculature asymmetry is a poor predictor of asymmetries elsewhere in the body. In addition, multiple asymmetries were uncommon and seemed closely related to the remote islands’ endogamous populations. As much as 72% of all subjects who had multiple asymmetries originated from the island of Vis, as opposed to 6.5% from the city of Split. However, after adjusting for various confounding factors, the model indicated that the presence of multiple asymmetries did not exert a direct impact on survival.

The fact that the results lack significance in either ReVA or multiple asymmetries in terms of survival is rather interesting. Experimental studies in animals often resonate with the theoretical assumptions of better health and fitness in more symmetric organisms, an effect that is often reported as more pronounced in males [39]. The stratified analysis results in this study did not support any sex-based difference, suggesting that, in both men and women, ReVA was an insignificant predictor of survival.

Interestingly, we also showed that ROHs did not contribute significantly to the survival model. The lack of significance of both asymmetry and ROHs in the prediction of survival is interesting, but also expected, confirming a previous finding that ROHs did not predict survival to old age in another human isolate sample [40]. Notably, there may be problems related to these calculations, reflecting the accompanying change in the declining ROHs in modern populations [21,41], coupled with changes in lifestyle patterns that may additionally affect mortality risks [42,43]. Such a situation may lead to a balance of the effects of risk factors towards net mortality, requiring careful interpretation in the context of constant changes.

The results of this study suggest that the existence of asymmetries does not seem to cluster substantially, and that each organ or tissue might be experiencing a local asymmetry risk. This implies the existence of organ-specific mechanisms of asymmetry that operate autonomously, potentially exhibiting varying thresholds for genetic and environmental perturbations. This is in line with previous studies that suggest that ciliary movement during neurulation may have a central role in asymmetry [44,45], meaning that the organ composition may contribute to asymmetry and may indeed be organ-specific rather than organism-specific.

The limitations of this study include a comparatively small sample size due to incomplete data (especially the audiometry estimates). There is an inherent risk of selection bias, as is present in any population-based study, which increases the risk of the most interesting subjects being missed and suffers from the oversampling of healthier and wealthier strata of the population. Any genetic study in an isolated population is hindered by the locally specific genetic structure, which might not be readily generalisable elsewhere. Nevertheless, the results of this study can contribute to a wider understanding of body asymmetry in an attempt to better understand human health and disease.

## Figures and Tables

**Table 1:** Demographics and basic measurements of the three analysed sub-cohorts.

Parameter	Island of Vis	Island of Korcula	City of Split	Total	*p*
Sex; *n* (%) Men Women					0.564
	237 (38.4)	195 (41.5)	307 (39.1)	739 (39.5)	
	380 (61.6)	275 (58.5)	479 (60.9)	1134 (60.5)	
Comorbidity One Multiple None					<0.001
	185 (30.0)	121 (25.7)	209 (26.6)	515 (27.5)	
	171 (27.7)	123 (26.2)	92 (11.7)	386 (20.6)	
	261 (42.3)	226 (48.1)	485 (61.7)	972 (51.9)	
Age	55.63 ± 14.22	54.47 ± 15.62	51.04 ± 14.07	53.41 ± 14.66	<0.001
Years of schooling	10.04 ± 3.42	10.53 ± 3.32	13.09 ± 2.89	11.40 ± 3.48	<0.001
Subjective material status	2.98 ± 0.76	3.12 ± 0.71	3.32 ± 0.69	3.16 ± 0.74	<0.001
Objective material status	*	3.24 ± 1.43	4.16 ± 1.4	3.82 ± 1.48	<0.001
Household material status	9.53 ± 2.65	10.22 ± 2.44	11.3 ± 2.34	10.45 ± 2.59	<0.001

* not available in this cohort.

**Table 2:** The selection of significant correlations of the ReVA and asymmetry measurements elsewhere in the body.

	ReVA.0; r (*p*)	ReVA.1; r (*p*)
ReVA.0	-	0.994 (<0.001)
ReVA.1	0.994 (<0.001)	-
Spheric power	0.133 (<0.001)	0.135 (<0.001)
Cylinder power	0.163 (<0.001)	0.164 (<0.001)
Angle	0.042 (0.135)	0.045 (0.117)
Corneal radius	0.124 (<0.001)	0.123 (<0.001)
Corneal thickness	0.082 (<0.001)	0.085 (<0.001)
Anterior chamber length	0.189 (<0.001)	0.193 (<0.001)
Lens thickness	0.084 (<0.001)	0.087 (<0.001)
Posterior chamber length	0.165 (<0.001)	0.169 (<0.001)
Axial length	0.191 (<0.001)	0.197 (<0.001)
Audiometry, 0.25 Hz	0.011 (0.641)	0.007 (0.757)
Audiometry, 0.5 Hz	0.030 (0.199)	0.031 (0.192)
Audiometry, 1 Hz	0.033 (0.165)	0.031 (0.184)
Audiometry, 2 Hz	−0.009 (0.701)	−0.006 (0.788)
Audiometry, 3 Hz	0.035 (0.131)	0.037 (0.110)
Audiometry, 4 Hz	0.054 (0.020)	0.050 (0.032)
Audiometry, 6 Hz	−0.004 (0.856)	−0.005 (0.822)
Audiometry, 8 Hz	0.052 (0.028)	0.052 (0.025)
ABPI, radial artery	0.034 (0.175)	0.032 (0.200)
ABPI posterior tibial artery	0.101 (<0.001)	0.097 (<0.001)
ABPI dorsal foot artery	0.034 (0.176)	0.033 (0.181)
ROH, number of segments	0.229 (<0.001)	0.229 (<0.001)
ROH, kb	0.101 (<0.001)	0.099 (<0.001)
ROH, average kb	0.098 (<0.001)	0.100 (<0.001)
ROH, number of segments, rLD	0.213 (<0.001)	0.213 (<0.001)
ROH, kb, rLD	0.089 (<0.001)	0.088 (<0.001)
ROH, average kb, rLD	0.108 (<0.001)	0.111 (<0.001)

**Table 3:** Results of the Cox regression model of survival, showing the hazard ratios adjusted for the confounder effects.

Variable	Unadjusted Model, HR [95% CI]; *p*	Adjusted Model, HR [95% CI]; *p*
Age	1.10 [1.09–1.12]; <0.001	1.09 [1.08–1.11]; <0.001
Sex		
Men (Ref)	1.00	1.00
Women	0.53 [0.41–0.69]; <0.001	0.49 [0.37–0.64]; <0.001
Cohort		
Vis (Ref)	1.00	1.00
Korcula	0.87 [0.56–1.36]; 0.533	0.62 [0.36–1.04]; 0.069
Split	0.58 [0.41–0.82]; 0.002	0.88 [0.59–1.29]; 0.500
Multiple asymmetries		
No (Ref)	1.00	1.00
Yes	0.97 [0.59–1.59]; 0.899	0.74 [0.45–1.22]; 0.237
Comorbidity load	1.64 [1.49–1.80]; <0.001	1.20 [1.08–1.35]; 0.002
ROH, number of segments	1.02 [1.00–1.05]; 0.031	0.99 [0.97–1.02]; 0.789

## Data Availability

Raw data for this study are available upon reasonable request from the corresponding author.
